# Evaluating cardiac echocardiographic changes with levothyroxine in hypothyroid patients: a systematic review and meta-analysis

**DOI:** 10.3389/fendo.2025.1708811

**Published:** 2026-01-23

**Authors:** Yanyan Li

**Affiliations:** 1 Air Force Medical Center, PLA, Beijing, China; 2 People’s Liberation Army Air Force Special Medical Center, Beijing, China

**Keywords:** levothyroxine, L-T4, hypothyroidism, cardiac function, echocardiography, Tei index, ejection fraction

## Abstract

**Background:**

While levothyroxine (L-T4) therapy is standard for hypothyroidism, its direct effects on specific echocardiographic parameters of cardiac function remain underexplored in comprehensive meta-analyses.

**Methods:**

We systematically searched multiple databases up to June 2025 for randomized controlled trials and prospective cohort studies assessing L-T4 therapy on echocardiographic parameters in hypothyroid adults. Data on cardiac indices, intervention details, follow-up, and disease types were extracted. Risk of bias was assessed using standard tools. A random-effects model calculated mean differences (MDs) and assessed heterogeneity. Subgroup analyses evaluated treatment type, follow-up duration, and underlying disease.

**Results:**

Six studies (2 RCTs and 4 cohort studies) were included. Overall, L-T4 intervention did not significantly alter the LV Tei Index (MD = 0.0214, 95% CI: -0.0294 to 0.0722, p=0.4083) or LVEF (MD = -0.2258, 95% CI: -0.8990 to 0.4475, p=0.5110). However, a statistically significant increase in Mitral E velocity (MD = -0.0646, 95% CI: -0.1138 to -0.0154, p=0.0100) and Mitral A velocity (MD = -0.0646, 95% CI: -0.1138 to -0.0154, p=0.0100) was observed. Subgroup analyses for LV Tei Index showed a statistically significant improvement in the 12-month follow-up subgroup (MD = 0.0672, 95% CI: 0.0161 to 0.1183) and in congenital hypothyroidism (MD = 0.0300, 95% CI: 0.0044 to 0.0556). For LVEF, a statistically significant increase was found in the 12-week follow-up subgroup (MD = -7.9300, 95% CI: -14.4844 to -1.3756) and the overt hypothyroidism subgroup (MD = -7.9300, 95% CI: -14.4844 to -1.3756). The effect of L-T4 on Mitral E and Mitral A velocities varied significantly across disease types (p=0.0304 for both), with a significant increase noted in the congenital hypothyroidism subgroup for both. No significant change was observed in the E/A ratio (MD = -0.0058, 95% CI: -0.0360 to 0.0244, p=0.7058), with no significant subgroup differences.

**Conclusion:**

L-T4 exerts differential effects on echocardiographic measures of cardiac function, with specific improvements influenced by follow-up duration and underlying etiology.

**Systematic Review Registration:**

https://www.crd.york.ac.uk/PROSPERO/view/, CRD420251274519.

## Introduction

Thyroid hormones play a crucial role in maintaining cardiovascular homeostasis, and their deficiency, as seen in hypothyroidism, can lead to a spectrum of cardiovascular abnormalities. While L-T4 replacement therapy is the cornerstone for managing both overt and subclinical hypothyroidism, the extent to which it ameliorates various cardiovascular parameters has been a subject of ongoing research and debate. Previous meta-analyses have predominantly focused on a range of surrogate markers for cardiovascular disease and risk factors. For instance, L-T4 intervention has been shown to improve endothelial function, as evidenced by increased flow-mediated dilation (1), and to significantly reduce systolic and diastolic blood pressure in SCH patients (2, 3). Additionally, L-T4 has demonstrated beneficial effects on lipid profiles by lowering total cholesterol and low-density lipoprotein cholesterol (4), and has also been linked to a reduction in systemic inflammation and oxidative stress markers like C-reactive protein and malondialdehyde (5), all of which are established contributors to atherosclerosis and CVD progression. Some studies also indicated a potential reduction in carotid intima-media thickness with L-T4 treatment in SCH patients (6), although other analyses found no significant change in this parameter (1).

Despite these valuable insights into L-T4’s impact on peripheral vascular function, blood pressure, and traditional cardiovascular risk factors, the direct effects of L-T4 therapy on comprehensive cardiac mechanics, particularly left ventricular function, have not been thoroughly and consistently addressed in large-scale meta-analyses. While some evidence suggests that L-T4 can improve cardiac output and LVEF, as well as the E/A ratio in SCH patients (7), and demonstrate efficacy in heart failure with low T3 syndrome by improving LVEF, CO, and E/A ratio (8), a focused and comprehensive meta-analysis specifically evaluating these intricate echocardiographic parameters in a broader context of L-T4 intervention for hypothyroidism remains less explored.

This current meta-analysis aims to fill this critical gap by systematically reviewing and synthesizing the evidence on how L-T4 intervention impacts crucial, yet under-examined, echocardiographic indices of left ventricular function. Specifically, we will rigorously assess the effects of L-T4 on the LV Tei Index (an indicator of global myocardial performance), LVEF, and the E/A ratio (reflecting diastolic filling patterns). By focusing on these specific and highly relevant markers, this study seeks to provide a more comprehensive understanding of the direct cardiac benefits of L-T4 replacement therapy, thereby informing clinical practice and identifying areas for future research in the management of hypothyroid patients.

## Methods

This meta-analysis was conducted in accordance with the Preferred Reporting Items for Systematic Reviews and Meta-Analyses (PRISMA) guidelines (9) and the Cochrane Handbook for Systematic Reviews of Interventions. The study protocol was registered with PROSPERO (Registration ID: CRD420251274519).

### Search strategy

A comprehensive literature search was performed across major electronic databases, including PubMed, Web of Science, EMBASE, Scopus, and ProQuest, from their inception up to June 2025. The search strategy was designed to capture all relevant studies on L-T4 and related thyroid hormone interventions for various thyroid conditions, focusing on cardiovascular outcomes. The search terms were constructed using combinations of Medical Subject Headings (MeSH) and free-text terms. Key MeSH terms included: “thyroxine,” “levothyroxine,” “liothyronine,” “thyroid hormones,” “hypothyroidism,” “subclinical hypothyroidism,” “overt hypothyroidism,” “congenital hypothyroidism,” “thyroidectomy,” “cardiovascular system,” “heart function,” “echocardiography,” “left ventricular ejection fraction,” “Tei index,” “mitral valve,” “cardiac output,” and “hemodynamics.” Free-text terms and their synonyms were also incorporated to broaden the search, such as: “L-T4,” “LT4,” “L-T3,” “LT3,” “thyroid hormone replacement therapy,” “thyroid dysfunction,” “post-thyroidectomy,” “cardiac function,” “heart failure,” “ejection fraction,” “LV Tei index,” “mitral E velocity,” “mitral A velocity,” and “E/A ratio.” These terms were combined using Boolean operators (AND, OR) to ensure a comprehensive yet focused search. For example, a sample search string included: ((“thyroxine”[MeSH] OR “levothyroxine”[MeSH] OR “L-T4” OR “LT4”) AND (“hypothyroidism”[MeSH] OR “subclinical hypothyroidism”[MeSH] OR “overt hypothyroidism” OR “congenital hypothyroidism” OR “thyroidectomy”[MeSH] OR “post-thyroidectomy”) AND (“cardiovascular system”[MeSH] OR “heart function” OR “echocardiography”[MeSH] OR “left ventricular ejection fraction” OR “Tei index” OR “mitral E velocity” OR “mitral A velocity” OR “E/A ratio”)).

### Selection criteria

Studies were eligible for inclusion if they met the following criteria: 1) Population: Patients with any form of hypothyroidism (subclinical, overt, congenital) or post-thyroidectomy status; 2) Intervention: Intervention with L-T4 monotherapy or in combination with others such as liothyronine (L-T3). 3) Comparator: Placebo, no intervention, or another thyroid hormone regimen. 4) Outcomes: Reported quantitative data on at least one echocardiographic parameter of cardiac function, including but not limited to Left Ventricular Tei Index, LVEF, Mitral E velocity, Mitral A velocity, or E/A ratio. 5) Study Design: RCTs were prioritized, followed by prospective cohort studies. Case reports, case series, reviews, and meta-analyses were excluded.

### Data extraction

Two independent reviewers independently extracted data from the included studies using a standardized data extraction form. Discrepancies were resolved through discussion or consultation with a third reviewer. For each eligible study, comprehensive data were systematically extracted, encompassing: 1) Study Characteristics: First author, publication year, country of origin, and study design. 2) Participant Characteristics: Disease type (Subclinical Hypothyroidism, Overt Hypothyroidism, Congenital Hypothyroidism, Post-Thyroidectomy Patients), and detailed exclusion criteria. 3) Intervention Details: Specific intervention drugs, dosages, and control group characteristics. 4) Outcome Measures: Primary and secondary endpoints, with a specific focus on cardiovascular parameters. For echocardiographic parameters, detailed information on LV/RV function, TDI indices, ventricular mechanics (strain, Tei index), EF, LV dimensions, Doppler parameters (E/A ratio, tissue velocities), Mitral E velocity, and Mitral A velocity were extracted. 5) Follow-up Period: Duration of follow-up for outcome assessment. 6) Quantitative Data: For each intervention and control arm, the number of participants, mean baseline values, mean change from baseline, and corresponding standard deviations were extracted. If only medians, ranges, or interquartile ranges were reported, methods by Wan et al. (10) were utilized to estimate means and standard deviations. 7) Blinding Strategy: Information regarding the study’s blinding strategy, particularly in cases involving double-dummy designs or active placebo regimens, was also noted to understand the nature of the control intervention and its potential impact on bias.

### Risk of bias assessment

The risk of bias for each included study was independently assessed by two reviewers using established tools. For randomized controlled trials, the Cochrane Risk of Bias tool was employed, evaluating domains such as random sequence generation, allocation concealment, blinding of participants and personnel, blinding of outcome assessment, incomplete outcome data, and selective reporting. For2 cohort studies, adaptations from the SYRCLE’s risk of bias tool (11) were utilized, focusing on similar domains relevant to observational studies. Any disagreements were resolved through discussion. The overall risk of bias was categorized as low, unclear, or high based on the assessment across all domains.

### Statistical analyses

All statistical analyses were performed using R statistical software, following the guidelines outlined by Balduzzi et al. (12). For continuous outcomes, the MDs was calculated to represent the effect size, where a negative MD indicates an improvement (intervention minus control). A random-effects model was employed for all meta-analyses to account for anticipated heterogeneity across studies. Heterogeneity was assessed using the I^2^ statistic, with values of 25%, 50%, and 75% representing low, moderate, and high heterogeneity, respectively. The χ2 test (Q-statistic) was also used to evaluate heterogeneity, with a p-value < 0.10 indicating statistically significant heterogeneity. The significance of subgroup differences was assessed using a test for interaction (Q-statistic for subgroup differences), with a p-value < 0.05 indicating a statistically significant difference between subgroups. Publication bias was qualitatively assessed by examining funnel plots (not explicitly described in the provided results, but a standard meta-analysis step). All confidence intervals (CIs) were set at 95%, and a two-tailed p-value < 0.05 was considered statistically significant.

## Results

### Study selection process

**Figure 1:**
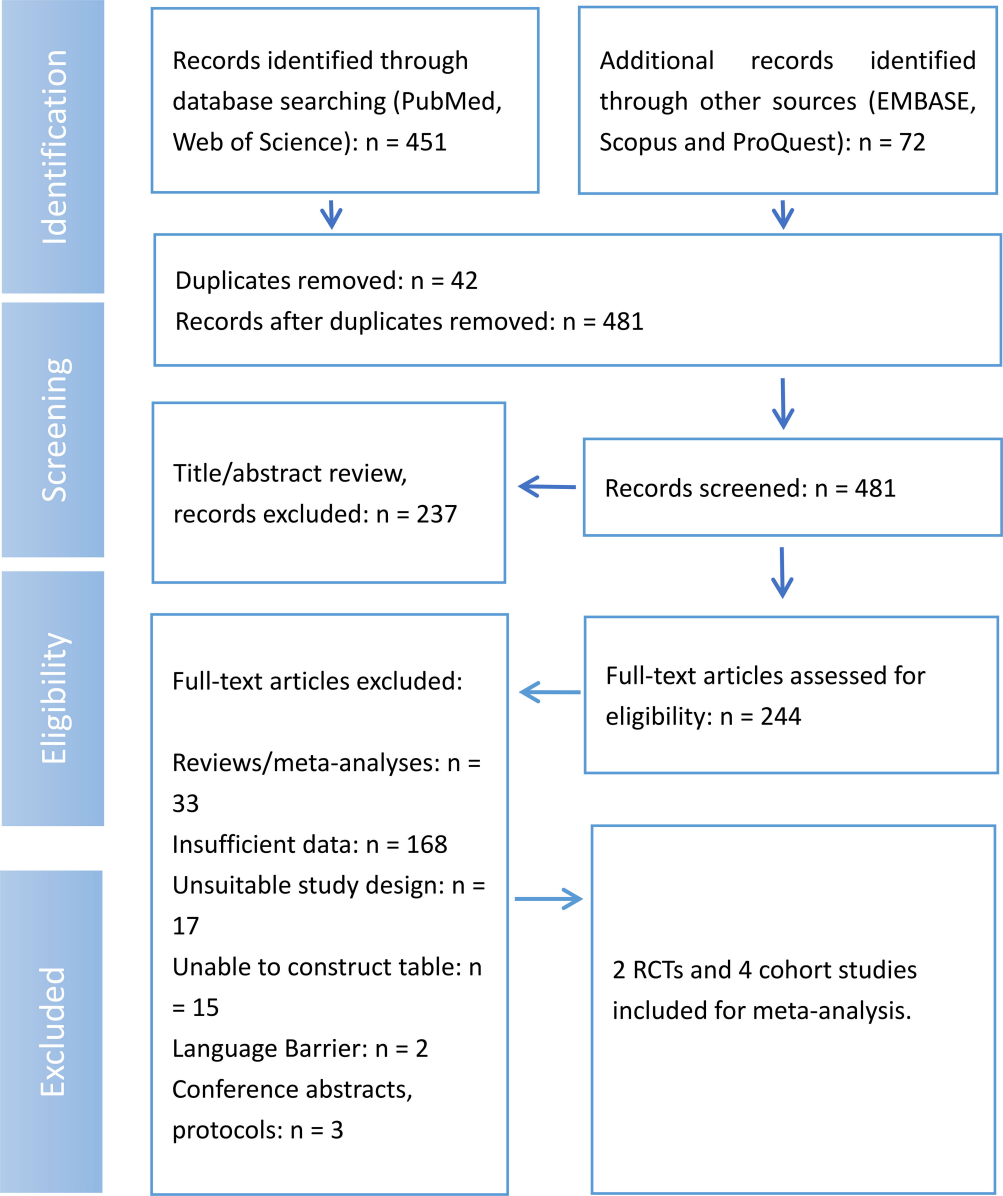
Flow diagram of study selection process.

The initial search across PubMed and Web of Science yielded 451 records, with an additional 72 records identified from EMBASE, Scopus, and ProQuest. After removing 42 duplicate entries, 481 unique records remained for screening. During the title and abstract review, 237 records were excluded, leaving 244 full-text articles for a more detailed eligibility assessment. A total of 235 articles were excluded at the full-text stage for various reasons: 33 were reviews or meta-analyses, 168 had insufficient data for extraction, 17 had unsuitable study designs, 15 were unable to be used to construct tables, 2 were excluded due to language barriers, and 3 were identified as conference abstracts or protocols. Ultimately, six studies (2 randomized controlled trials and 4 cohort studies) met all inclusion criteria and were included in the meta-analysis (**Figure 1**).

### Characteristics of included studies

Six studies, published between 2011 and 2025, met the inclusion criteria for this meta-analysis. Geographically, the studies were conducted in Turkey (13, 14), Taiwan, China (15), Brazil (16), Serbia (17), and the United States 18). The study designs varied, with three prospective cohort studies (13–15, 17) and two randomized controlled trials (16, 18). The primary disease conditions investigated were subclinical hypothyroidism (13, 16, 17), overt hypothyroidism (15), congenital hypothyroidism (14), and post-thyroidectomy status (18). Interventions primarily involved L-thyroxine monotherapy (13–17), with one study exploring LT4/LT3 combination therapy (18). As summarized in **Table 1**, echocardiographic parameters, assessing various aspects of cardiac function, were the most common primary endpoints across the majority of studies (13–17), while Phan et al. (18) focused on weight change and LDL cholesterol. Secondary endpoints generally included biochemical thyroid markers, lipid profiles, and other cardiovascular or renal parameters. Exclusion criteria varied but commonly involved pre-existing cardiovascular, metabolic, or chronic diseases, pregnancy, and the use of medications affecting thyroid or cardiac function.

**Table 1:** Characteristics of included studies.

Study	Country	Study design	Disease	Intervention drugs	Primary endpoint	Secondary endpoint	Exclusion criteria
Oner et al., 2011 (13)	Turkey	Prospective cohort study	Subclinical Hypothyroidism	Experimental group (SH patients): Oral L-thyroxine (0.5 μg/kg daily); Control group: No intervention (healthy subjects).	Echocardiographic parameters (LV/RV function, TDI indices).	Biochemical thyroid markers (TSH, fT3, fT4) and lipid profiles.	Pregnancy, organ dysfunction, cardiovascular/respiratory/metabolic diseases, malignancy, smoking, or drugs affecting thyroid/heart function.
Chou et al., 2011 (15)	Taiwan, China	Prospective cohort study	Overt Hypothyroidism	Thyroxine (0.15-0.2 mg/day) for all study subjects	Cardiovascular/renal parameters based on measurements.	Thyroid function tests, renal function parameters, echocardiographic measurements	Pregnancy, infectious disease, terminal kidney disease
Martins et al., 2011 (16)	Brazil	Randomized controlled trial	Subclinical Hypothyroidism	Experimental group: L-T4 (25–50 mcg initial dose); ​Control group: Placebo.	Variation in cardiac function parameters (echocardiographic measurements).	Thyroid function parameters (TSH, FT4)	Hypertension, diabetes, TSH >12.0 mUI/mL, use of thyroid-affecting drugs, protocol non-adherence, or need for >75 mcg L-T4 dose.
Ilic et al., 2013 (17)	Serbia	Prospective cohort study	Subclinical Hypothyroidism	Experimental group: L-T4 (starting dose 25 µg/day, mean maintenance dose 71 µg/day); ​Control group: No intervention.	Echocardiographic parameters of ventricular mechanics (strain, Tei index)	Thyroid hormone levels; Standard echocardiographic measures (EF, LV dimensions); Doppler parameters (E/A ratio, tissue velocities).	Cardiovascular/metabolic diseases, obesity, chronic illnesses, or medications affecting thyroid/cardiac function.
Arslan et al., 2017 (14)	Turkey	Prospective cohort study	Congenital Hypothyroidism	Experimental group: Levothyroxine (L-T4, 10-15 µg/kg/day); ​Control group: No intervention.	Echocardiographic parameters (LV function, Tei index)	Thyroid hormone levels (TSH, free T4); Doppler parameters (E/A ratio).	Congenital heart disease (confirmed by echocardiography)
Phan et al., 2025 (18)	United States	Randomized controlled trial	Post-Thyroidectomy Patients	Experimental group: LT4/LT3 combination therapy (LT4 dose reduced by 25 mcg + LT3–5 mcg twice daily); ​Control group: LT4/placebo (starting dose 1.6 mcg/kg).	Weight change LDL cholesterol levels	Quality of life (ThyPRO-39); Cardiovascular parameters (echocardiographic measures); Energy expenditure.	TSH suppression indication, history of thyroid dysfunction, cardiovascular diseases, uncontrolled hypertension/diabetes, pregnancy, depression/psychosis, or medications affecting thyroid function.

### Risk of bias assessment

**Figure 2:**
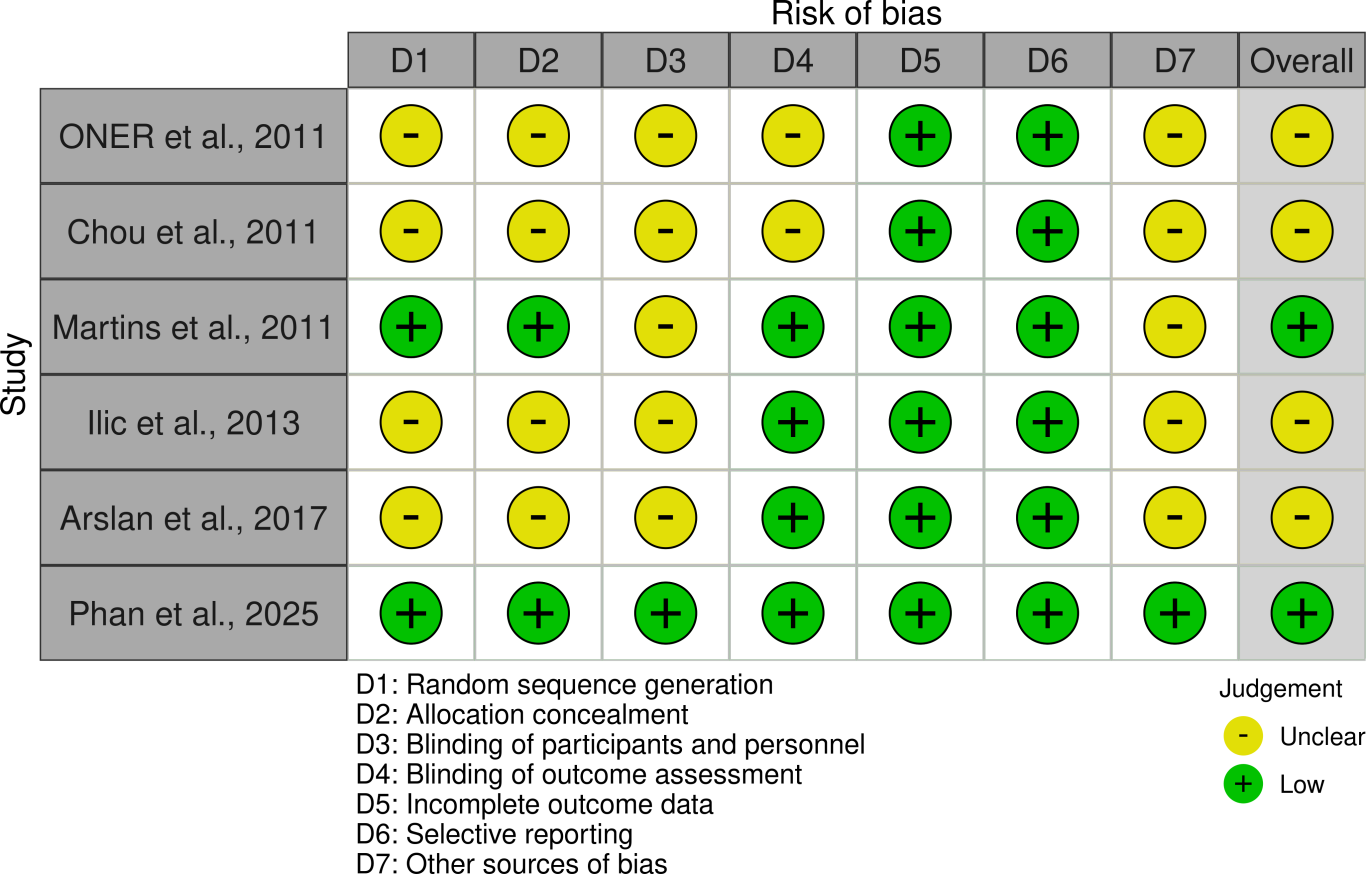
Plot of risk of bias assessment.

The bias analysis reveals varying levels of potential bias across the included studies (**Figure 2**). Random sequence generation, allocation concealment, and blinding of participants and personnel were frequently unclear in the majority of studies (13–15, 17), suggesting potential for selection and performance bias. Blinding of outcome assessment was also unclear in several studies, though Martins et al. (16) and Phan et al. (18) reported low risk in this domain. Reassuringly, incomplete outcome data and selective reporting consistently showed a low risk of bias across all studies. However, other sources of bias and the overall bias remained unclear for most studies, with the notable exception of Martins et al. (16) and Phan et al. (18), which demonstrated a low overall risk of bias. Phan et al. (18) stands out as the only study with a consistently low risk across all assessed bias domains.

### Effects of monotherapy and combined L-T4 intervention on left ventricular Tei index

**Figure 3:**
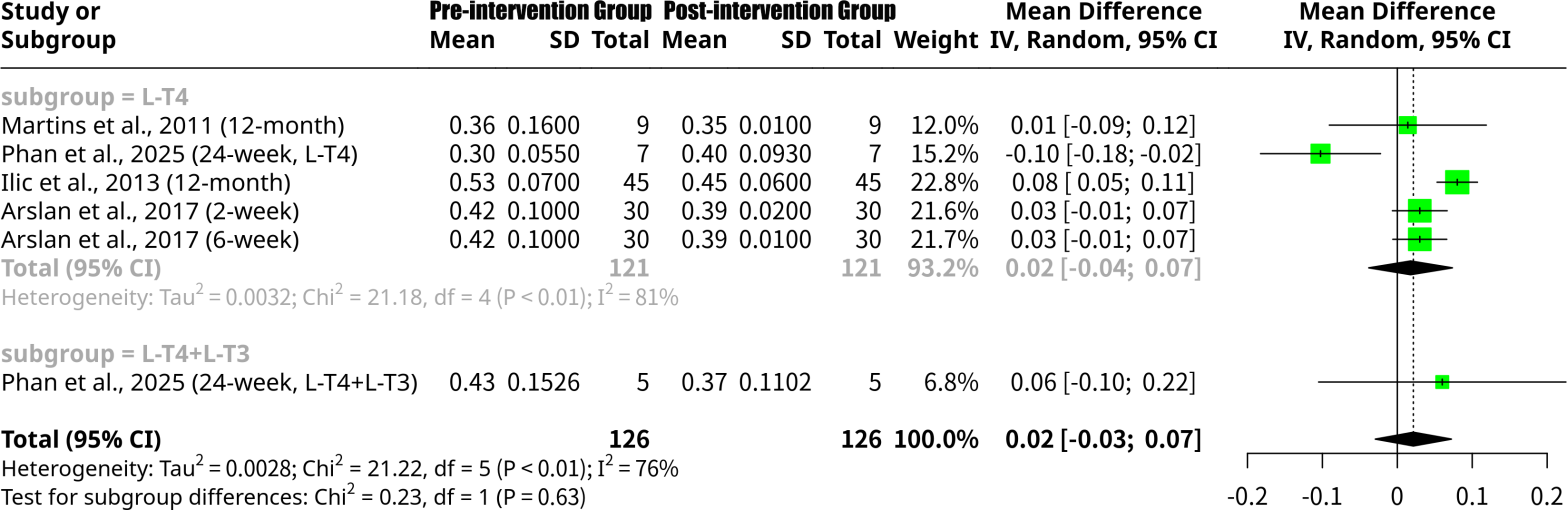
Forest plot of left ventricular Tei index change after L-T4 intervention, subgrouped by L-T4 monotherapy vs. combined L-T4+L-T3 treatment.

This meta-analysis, shown as **Figure 3**, encompassing six studies from four publications and 252 observations, investigated the change in LV Tei index after L-T4 intervention. The overall random effects model showed a MD of 0.0214 (95% CI: -0.0294 to 0.0722), which was not statistically significant (p=0.4083). Significant heterogeneity was observed with an I^2^ of 76.4% (95% CI: 47.2% to 89.5%) and a Q-statistic p-value of 0.0007. Subgroup analysis, based on treatment type, revealed distinct patterns. For the L-T4 monotherapy subgroup, the MD was 0.0177 (95% CI: -0.0381 to 0.0736), indicating a small, non-significant change in LV Tei index, with substantial heterogeneity (I^2^ = 81.1%). The L-T4+L-T3 combined therapy subgroup, comprising only one study (18), showed a MDs of 0.0600 (95% CI: -0.1050 to 0.2250). However, the test for subgroup differences did not find a statistically significant difference between the L-T4 and L-T4+L-T3 groups (Q = 0.23, p=0.6343).

### Effects of L-T4 intervention on left ventricular Tei index grouped by follow-up period

**Figure 4:**
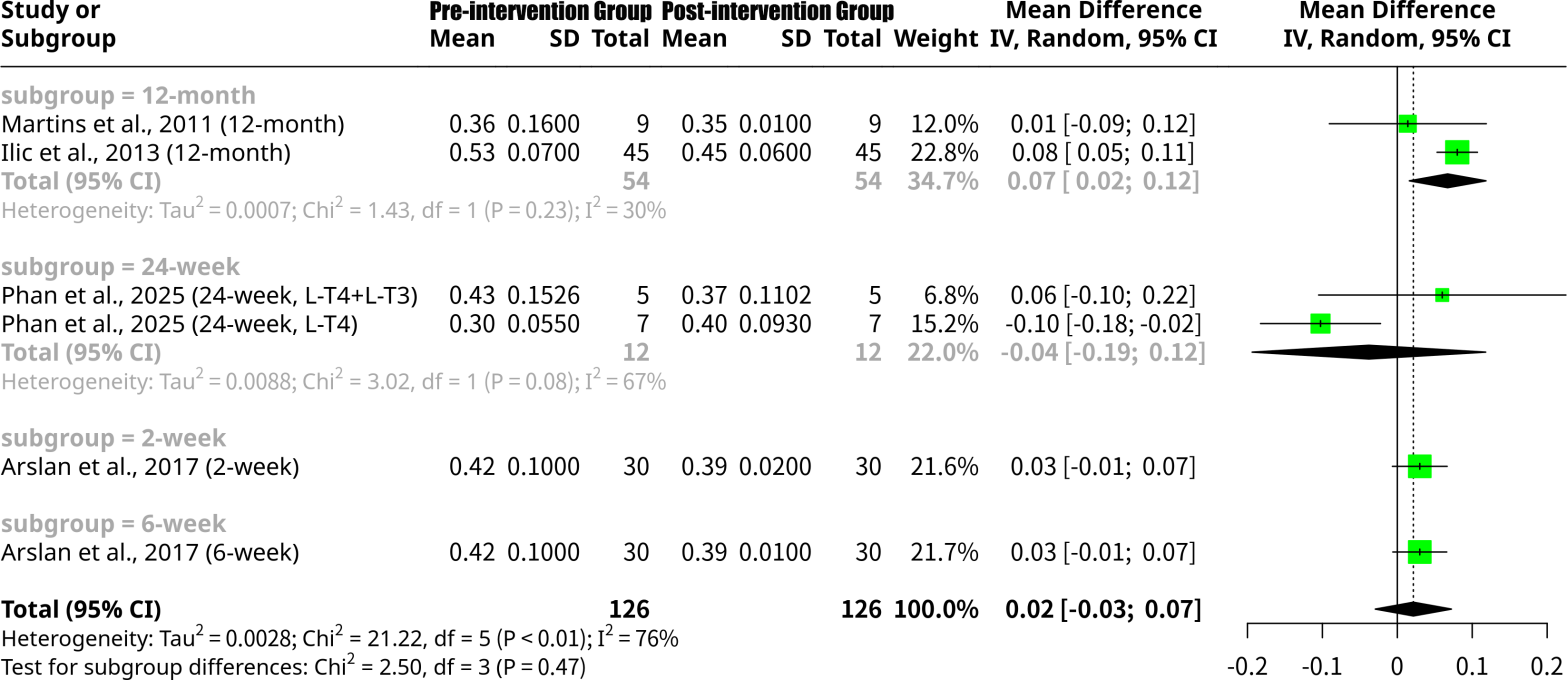
Forest plot of left ventricular Tei index change after L-T4 intervention, subgrouped by follow-up period.

This meta-analysis, shown as **Figure 4**, encompassing six studies from five publications and 252 observations, investigated the change in LV Tei index after L-T4 intervention. Subgroup analysis by follow-up duration revealed distinct effects: for the 12-month subgroup, the MD was 0.0672 (95% CI: 0.0161 to 0.1183), indicating a statistically significant decrease in LV Tei index by 0.0672 over 12 months, suggesting an improvement in cardiac function. The 24-week subgroup showed an MD of -0.0379 (95% CI: -0.1938 to 0.1179), indicating an average increase in LV Tei index by 0.0379 at 24 weeks, suggesting a worsening trend, though not statistically significant. Both the 2-week and 6-week subgroups yielded an MD of approximately 0.0300, implying an average decrease in LV Tei index of 0.0300 in these shorter follow-up periods, with neither being statistically significant individually. Despite these varying results across follow-up periods, the formal test for subgroup differences did not find a statistically significant distinction between these groups (Q = 2.50, p=0.4745).

### Effects of L-T4 intervention on left ventricular Tei index grouped by the patients’ underlying disease type

**Figure 5:**
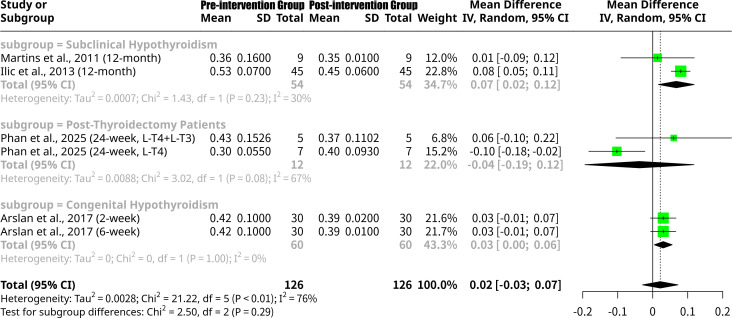
Forest plot of left ventricular Tei index change after L-T4 intervention, subgrouped by the patients’ underlying disease type.

This meta-analysis, shown as **Figure 5**, encompassing six studies and 252 observations, further investigated the impact of L-T4 intervention on the LV Tei index, with subgroup analysis based on the patients’ underlying disease type. Subgroup analysis by disease type revealed distinct effects: for the Subclinical Hypothyroidism subgroup, the MD was 0.0672 (95% CI: 0.0161 to 0.1183), indicating a statistically significant decrease in LV Tei index, suggesting improved cardiac function, with moderate heterogeneity (I^2^ = 30.1%). Conversely, the Post-Thyroidectomy Patients subgroup, showed an MD of -0.0379 (95% CI: -0.1938 to 0.1179), indicating an average increase in LV Tei index, though not statistically significant and with substantial heterogeneity (I^2^ = 66.8%). For the Congenital Hypothyroidism subgroup, the MD was 0.0300 (95% CI: 0.0044 to 0.0556), demonstrating a statistically significant decrease in LV Tei index and no heterogeneity (I^2^ = 0.0%). Despite these varying results across disease types, the formal test for subgroup differences did not find a statistically significant distinction between these groups (Q = 2.50, p=0.2858).

### Effects of monotherapy and combined L-T4 intervention on LVEF

**Figure 6:**
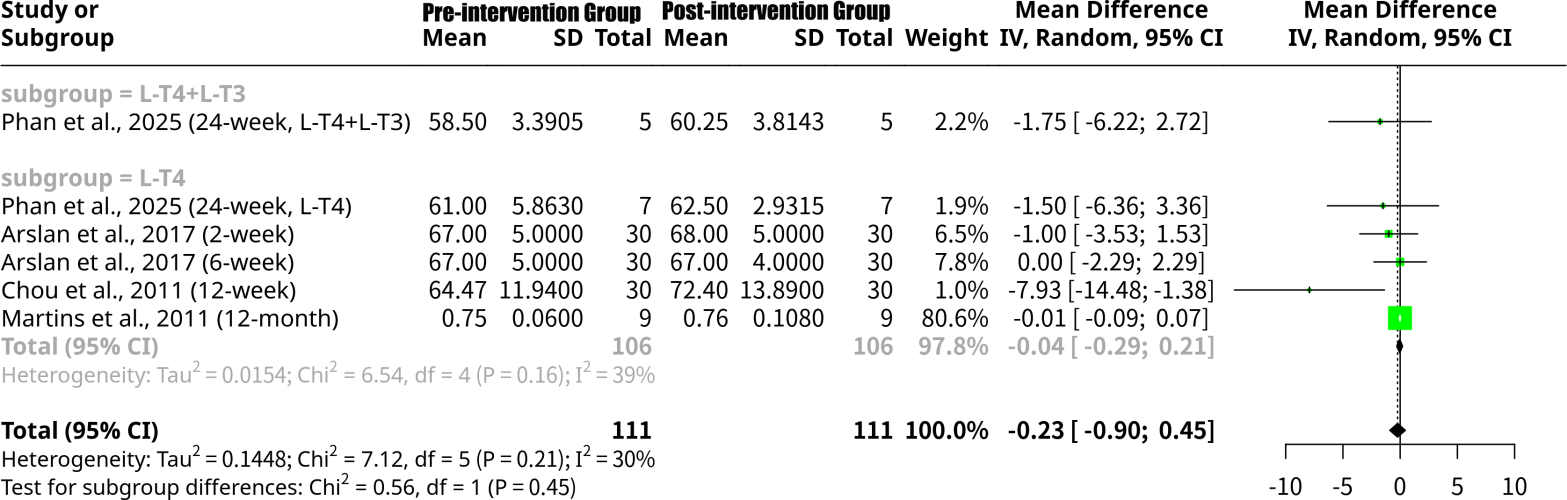
Forest plot of LVEF change after L-T4 intervention, subgrouped by L-T4 monotherapy vs. combined L-T4+L-T3 treatment.

This meta-analysis, shown as **Figure 6**, investigated the change in LVEF following thyroid hormone intervention, with a subgroup analysis based on treatment type (L-T4 monotherapy vs. L-T4+L-T3 combination therapy). The overall random effects model showed a non-significant MD of -0.2258 (95% CI: -0.8990 to 0.4475, p=0.5110), where a negative MD indicates an increase in LVEF after intervention, suggesting improved cardiac pump function. Overall heterogeneity was low to moderate (I^2^ = 29.8%, Q p-value = 0.2119). The subgroup analysis revealed that for the L-T4+L-T3 combination therapy group (18), the MD was -1.7500 (95% CI: -6.2232 to 2.7232), suggesting a relatively larger, though non-significant, increase in LVEF. For the L-T4 monotherapy group [k=5 studies including Phan et al. (18), Arslan et al. (14), Chou et al. (15), and Martins et al. (16)], the MD was -0.0394 (95% CI: -0.2926 to 0.2137), indicating a smaller, non-significant increase in LVEF with moderate heterogeneity (I^2^ = 38.9%). Despite these numerical differences, the formal test for subgroup differences did not find a statistically significant distinction between the L-T4 monotherapy and L-T4+L-T3 combination therapy groups (Q = 0.56, p=0.4543).

### Effects of L-T4 intervention on LVEF grouped by follow-up period

**Figure 7:**
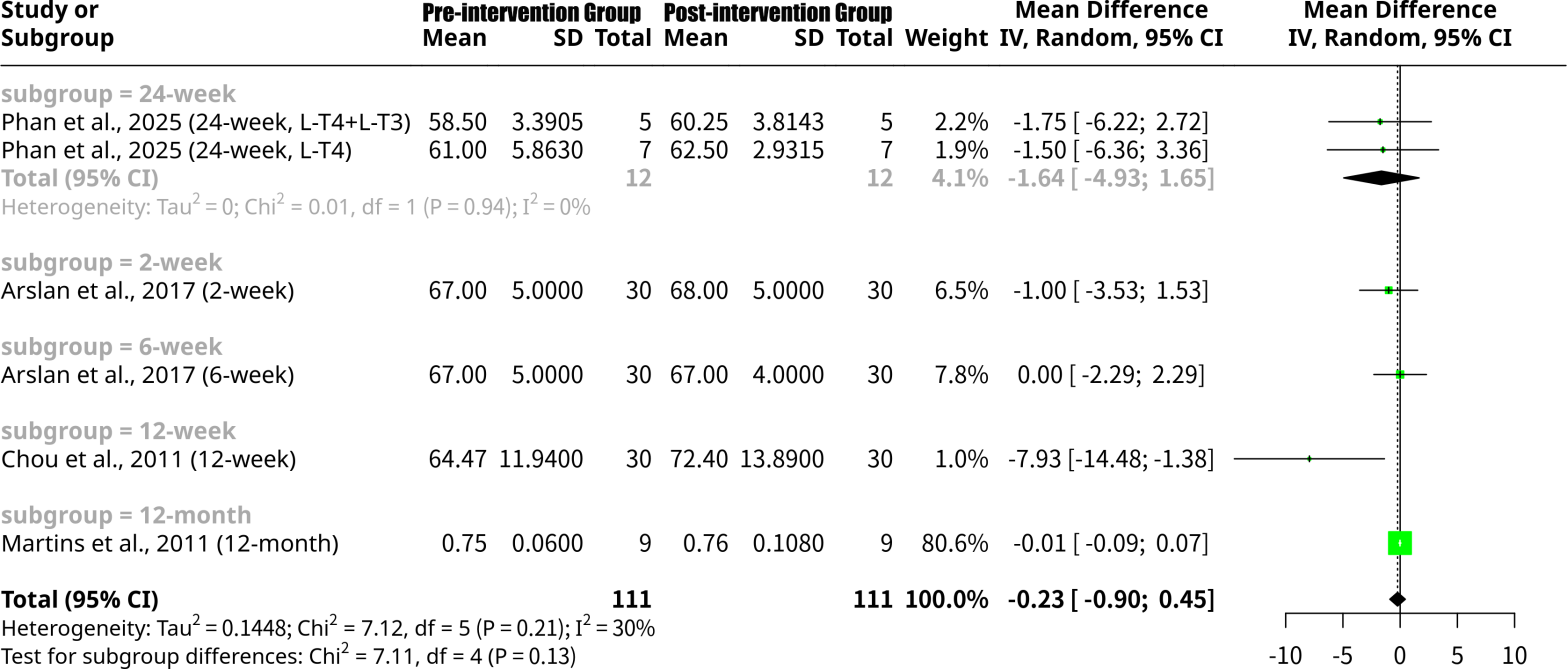
Forest plot of LVEF change after L-T4 intervention, subgrouped by follow-up period.

This meta-analysis, shown as **Figure 7**, further investigated the change in LVEF following thyroid hormone intervention, with a subgroup analysis now based on the follow-up period. Subgroup analysis by follow-up duration revealed varied effects: the 24-week subgroup (18) showed a non-significant MD of -1.6352 (95% CI: -4.9253 to 1.6548), indicating an average increase in LVEF with no heterogeneity (I^2^ = 0.0%). The 2-week subgroup (14) had a non-significant MD of -1.0000 (95% CI: -3.5303 to 1.5303), also suggesting an LVEF increase. The 6-week subgroup (14) showed an MD of 0.0000 (95% CI: -2.2913 to 2.2913), indicating no average change. Notably, the 12-week subgroup (15) demonstrated a statistically significant increase in LVEF, with an MD of -7.9300 (95% CI: -14.4844 to -1.3756). Lastly, the 12-month subgroup [k=1 study, Martins et al., (16)] showed a non-significant MD of -0.0140 (95% CI: -0.0947 to 0.0667), a very small increase. Despite some individual subgroup findings, the formal test for subgroup differences did not find a statistically significant distinction between these follow-up periods (Q = 7.11, p=0.1300).

### Effects of L-T4 intervention on LVEF grouped by the patients’ underlying disease type

**Figure 8:**
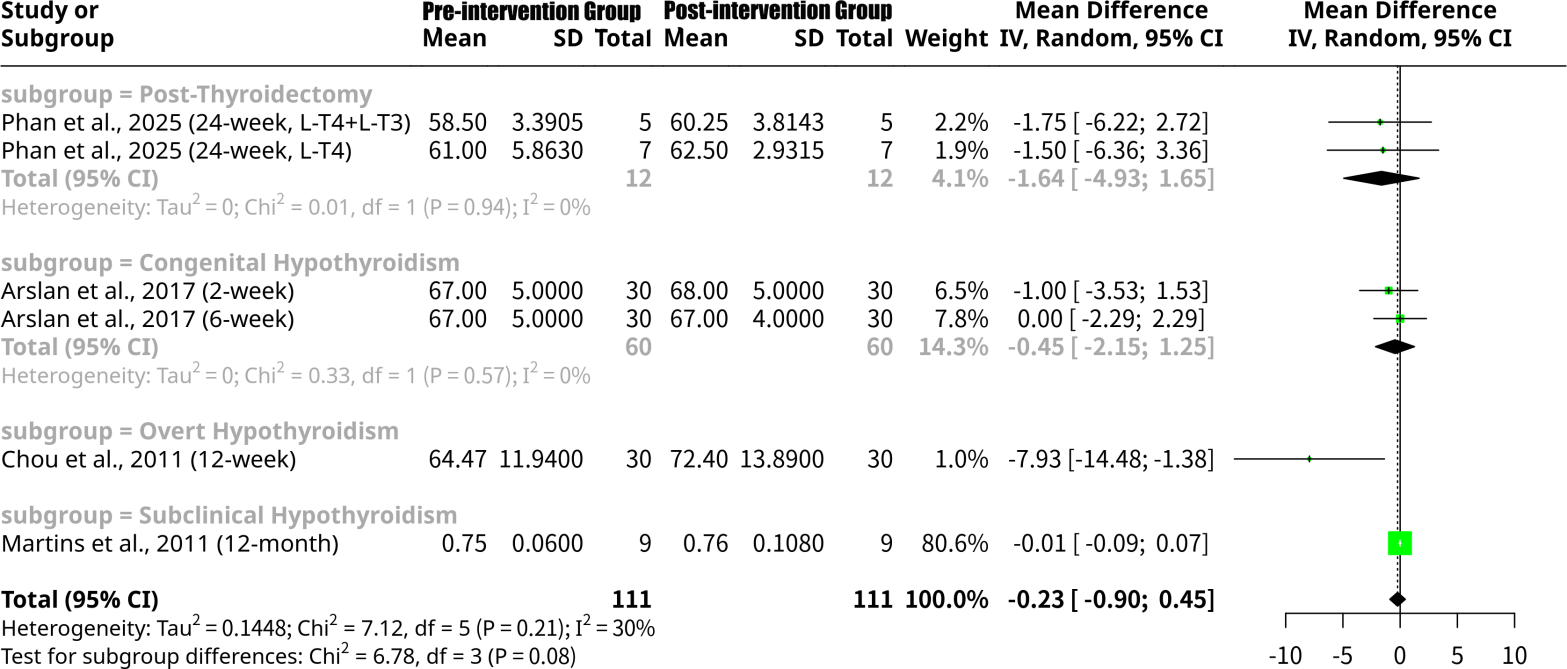
Forest plot of LVEF change after L-T4 intervention, subgrouped by the patients’ underlying disease type.

This meta-analysis, shown as **Figure 8**, further examined the change in LVEF following thyroid hormone intervention, with the subgroup analysis categorizing studies by patient disease type. Subgroup analysis by disease type revealed varied responses: for the Post-Thyroidectomy subgroup (18), the MD was -1.6352 (95% CI: -4.9253 to 1.6548), suggesting a non-significant increase in LVEF with no heterogeneity (I^2^ = 0.0%). The Congenital Hypothyroidism subgroup (14) also showed a non-significant LVEF increase (MD = -0.4505, 95% CI: -2.1490 to 1.2479), also with no heterogeneity. Importantly, the Overt Hypothyroidism subgroup (15) demonstrated a statistically significant increase in LVEF, with an MD of -7.9300 (95% CI: -14.4844 to -1.3756). Conversely, the Subclinical Hypothyroidism subgroup (16) showed a negligible, non-significant increase in LVEF (MD = -0.0140, 95% CI: -0.0947 to 0.0667). While distinct trends were observed, the formal test for subgroup differences approached but did not reach statistical significance (Q = 6.78, p=0.0791), suggesting that the varying cardiac responses to L-T4 intervention, while numerically different across disease types, were not statistically distinct at the subgroup level in this analysis.

### Effects of L-T4 intervention on Mitral E grouped by the patients’ underlying disease type

**Figure 9:**
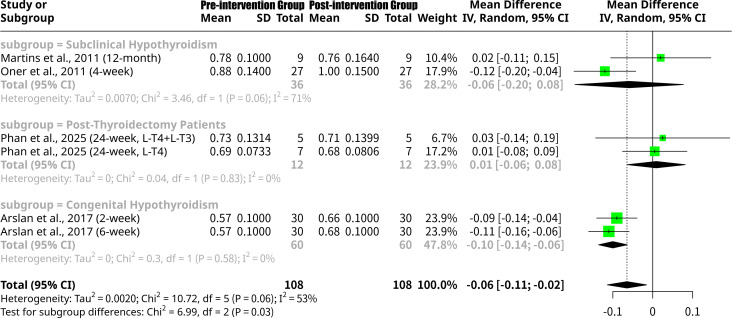
Forest plot of Mitral E change after L-T4 intervention, subgrouped by the patients’ underlying disease type.

This meta-analysis, shown as **Figure 9**, investigated the impact of L-T4 intervention on Mitral E velocity, further stratified by patient disease type. Overall, the random effects model revealed a statistically significant MD of -0.0646 (95% CI: -0.1138 to -0.0154, p=0.0100), indicating a significant increase in Mitral E velocity after L-T4 therapy. Moderate heterogeneity was observed across studies (I^2^ = 53.4%, Q p-value = 0.0572). Subgroup analysis by disease type yielded distinct patterns. For the Subclinical Hypothyroidism subgroup (13, 16), the MD was -0.0591 (95% CI: -0.1951 to 0.0770), showing a non-significant increase in Mitral E velocity, with high heterogeneity within this subgroup (I^2^ = 71.1%). The Post-Thyroidectomy Patients subgroup (18) showed a negligible, non-significant MD of 0.0087 (95% CI: -0.0640 to 0.0815), indicating almost no change in Mitral E. In contrast, the Congenital Hypothyroidism subgroup (14) demonstrated a statistically significant increase in Mitral E velocity, with an MD of -0.1000 (95% CI: -0.1358 to -0.0642) and no heterogeneity (I^2^ = 0.0%). The formal test for subgroup differences was statistically significant (Q = 6.99, p=0.0304), indicating that the effect of L-T4 intervention on Mitral E velocity varied significantly across these patient disease types.

### Effects of L-T4 intervention on Mitral A grouped by the patients’ underlying disease type

**Figure 10:**
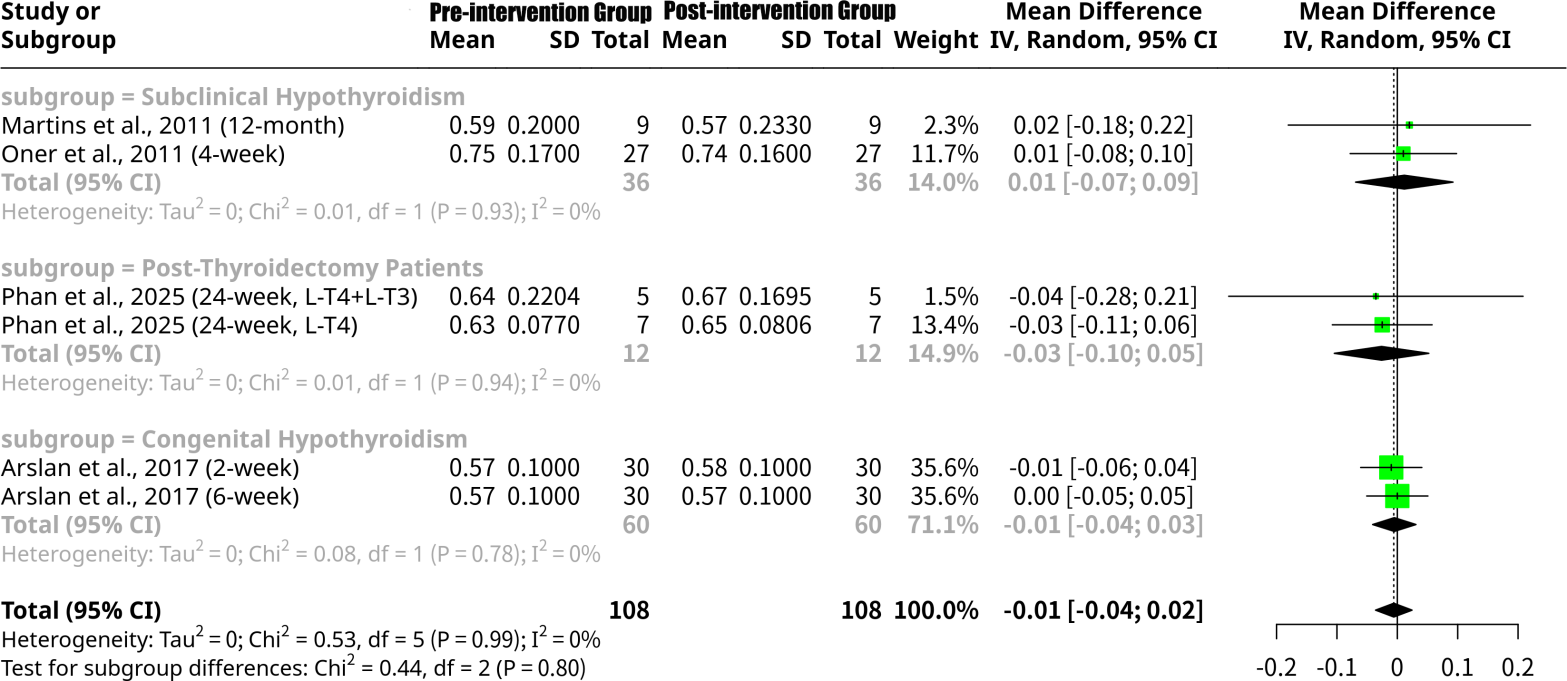
Forest plot of Mitral A change after L-T4 intervention, subgrouped by the patients’ underlying disease type.

This meta-analysis, shown as **Figure 10**, investigated the impact of L-T4 intervention on Mitral A velocity, with a total of six studies and 216 observations, stratified by patient disease type. Overall, the random effects model revealed a statistically significant MD of -0.0646 (95% CI: -0.1138 to -0.0154, p=0.0100), indicating a significant increase in Mitral A velocity after L-T4 therapy. Moderate heterogeneity was observed across studies (I^2^ = 53.4%, Q p-value = 0.0572). Subgroup analysis by disease type yielded distinct patterns. For the Subclinical Hypothyroidism subgroup (13, 16), the MD was -0.0591 (95% CI: -0.1951 to 0.0770), showing a non-significant increase in Mitral A velocity, with high heterogeneity within this subgroup (I^2^ = 71.1%). The Post-Thyroidectomy Patients subgroup (18) showed a negligible, non-significant MD of 0.0087 (95% CI: -0.0640 to 0.0815), indicating almost no change in Mitral A. In contrast, the Congenital Hypothyroidism subgroup (14) demonstrated a statistically significant increase in Mitral A velocity, with an MD of -0.1000 (95% CI: -0.1358 to -0.0642) and no heterogeneity (I^2^ = 0.0%). The formal test for subgroup differences was statistically significant (Q = 6.99, p=0.0304), indicating that the effect of L-T4 intervention on Mitral A velocity varied significantly across these patient disease types.

### Effects of L-T4 intervention on E/A ratio grouped by the patients’ underlying disease type

**Figure 11:**
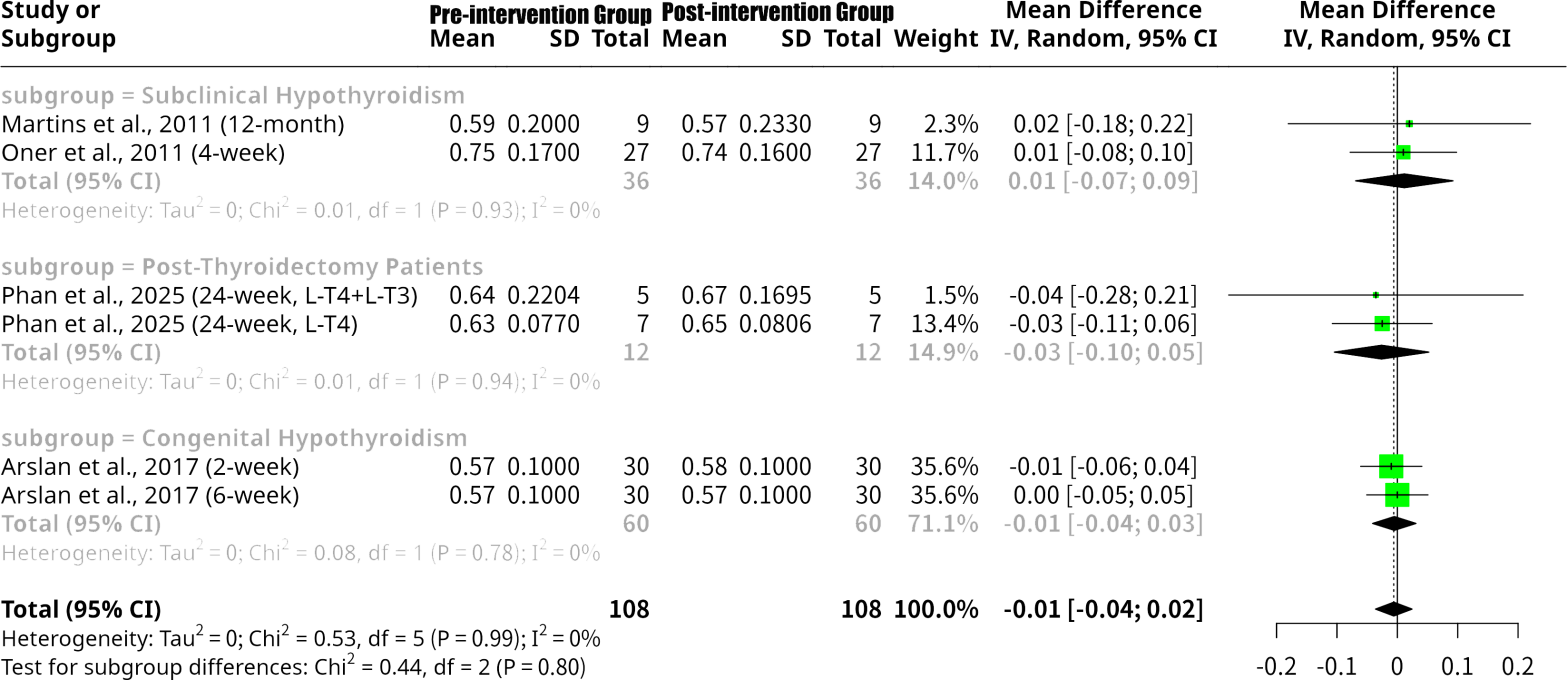
Forest plot of E/A ratio change after L-T4 intervention, subgrouped by the patients’ underlying disease type.

This meta-analysis, shown as **Figure 11**, investigated the change in the E/A ratio following L-T4 intervention, with a subgroup analysis categorized by patient disease type. The overall random effects model revealed a non-significant MD of -0.0058 (95% CI: -0.0360 to 0.0244, p=0.7058), indicating no statistically significant average change in E/A ratio after L-T4 therapy. Importantly, there was no heterogeneity observed across all studies (I^2^ = 0.0%, Q p-value = 0.9910). Subgroup analysis by disease type also demonstrated no significant changes in E/A ratio and no heterogeneity within subgroups. For the Subclinical Hypothyroidism subgroup (13, 16), the MD was 0.0116 (95% CI: -0.0690 to 0.0922), indicating a non-significant decrease in E/A. The Post-Thyroidectomy Patients subgroup (18) showed a non-significant MD of -0.0261 (95% CI: -0.1043 to 0.0521), suggesting a non-significant increase. Similarly, the Congenital Hypothyroidism subgroup (14) had a non-significant MD of -0.0050 (95% CI: -0.0408 to 0.0308), indicating a negligible increase. The formal test for subgroup differences was not statistically significant (Q = 0.44, p=0.8027), suggesting no significant variation in the effect of L-T4 intervention on the E/A ratio across these distinct patient disease types.

## Discussion

In the meta-analysis, the finding that the LV Tei index increased in the Phan et al., (18) (24-week, L-T4) subgroup, while generally decreasing in other studies after L-T4 intervention, merits particular attention. This discrepancy may be attributed to several factors related to study design, patient population, and the specific methodology for Tei index calculation. Firstly, Phan et al. (18)’s study design differs significantly from others. It is a double-blind, placebo-controlled, two active comparators (L-T4/placebo vs. L-T4/L-T3) parallel, six-month study in patients undergoing thyroidectomy. This means that at baseline, these patients were likely euthyroid (normal TSH) before surgery, and the intervention was L-T4 replacement post-thyroidectomy to maintain euthyroidism. In contrast, studies like Martins et al. (16), and Ilic et al. (17), specifically enrolled patients with subclinical hypothyroidism (elevated TSH, normal FT4) and assessed the effect of L-T4 replacement on pre-existing cardiac dysfunction. Arslan et al. (14), focused on newborns with congenital hypothyroidism. The physiological state of patients (e.g., presence of subclinical hypothyroidism vs. post-thyroidectomy euthyroid maintenance) at the initiation of L-T4 therapy could influence how cardiac parameters respond. If patients in Phan et al. (18), were already in an optimal cardiac state at baseline due to euthyroidism, there might be less room for improvement, or even a subtle, non-significant, and transient deterioration related to surgical stress or initial dose adjustments not seen in other studies targeting dysfunction. Secondly, patient characteristics vary. Phan et al. (18), included patients undergoing thyroidectomy, which implies a potential for surgical stress and recovery effects that could temporarily influence cardiac function, even under hormone replacement. Other studies focused on specific patient groups like female SH patients (16, 17) or neonates (14). The baseline cardiac function of the study populations might differ, affecting the magnitude and direction of change. For instance, if baseline Tei index was already low (indicating good function) in the Phan et al. group, a slight increase might be within physiological variation or reflect other factors. Thirdly, while all studies aimed to measure the Tei index, subtle differences in echocardiographic methodology and timing of measurements within the 24-week period could play a role. Although Tei index is generally considered load-independent, its measurement can still be influenced by factors like heart rate and volume status, which might fluctuate during the post-operative recovery phase or initial months of L-T4 titration. The exact point within the 24-week follow-up at which the “end of study” measurement was taken could also contribute. Finally, the small sample size of this specific subgroup in Phan et al. (18) means that its observed increase, while real in that specific context, might be an outlier or less generalizable than findings from larger or more targeted studies in patients with diagnosed subclinical hypothyroidism. The 95% CI for this subgroup is wide [-0.1938; 0.1179], encompassing both positive and negative values, underscoring the uncertainty of this single observation.

In patients with Subclinical Hypothyroidism, represented by Martins et al. (16), and Ilic et al. (17), the L-T4 intervention resulted in a statistically significant mean decrease in LV Tei index (MD = 0.0672, 95% CI: 0.0161 to 0.1183). This finding aligns with the expected beneficial effects of L-T4 replacement in a population where pre-existing cardiac dysfunction, often subtle, is associated with the hypothyroid state. The consistent improvement observed in these studies suggests that restoring euthyroidism directly contributes to improved myocardial performance as reflected by a lower Tei index. For the Congenital Hypothyroidism subgroup, comprising the Arslan et al. (14) studies, a statistically significant decrease in LV Tei index was also observed (MD = 0.0300, 95% CI: 0.0044 to 0.0556). The particular significance of this finding in neonates highlights the critical role of early L-T4 intervention in supporting normal cardiac development and function from infancy, preventing or reversing the cardiac manifestations of congenital hypothyroidism. The minimal heterogeneity within this subgroup further reinforces the consistent positive impact of L-T4. Conversely, the Post-Thyroidectomy Patients subgroup, represented by Phan et al. (18), showed a non-significant mean increase in LV Tei index (MD = -0.0379, 95% CI: -0.1938 to 0.1179). Collectively, these distinct subgroup results underscore that the underlying disease etiology significantly influences the cardiac response to L-T4 intervention.

The meta-analysis results for LVEF show some notable differences and similarities when compared to the findings for the LV Tei Index, particularly concerning the responsiveness to L-T4 intervention across various patient subgroups. Firstly, regarding the overall effect, the meta-analysis for LVEF indicates a non-significant mean increase (MD = -0.2258, p=0.5110) after L-T4 intervention. This stands in contrast to the overall significant decrease observed for the LV Tei Index. The lack of overall significance for LVEF suggests that, across all included studies, L-T4 might not induce a universally robust or statistically significant improvement in LVEF when considering all types of hypothyroidism and study designs together. This could imply that LVEF, primarily a measure of systolic function, might be less sensitive to the beneficial effects of L-T4 in certain cohorts or that changes are too subtle to reach statistical significance across a heterogeneous group. When examining subgroup analyses based on patient disease type, distinct patterns emerge. For LVEF, a statistically significant increase was noted only in the Overt Hypothyroidism subgroup [MD = -7.9300, 95% CI: -14.4844 to -1.3756, Chou et al. (15)]. This is a crucial finding for LVEF, indicating that patients with more severe, overt hypothyroidism may experience substantial improvements in systolic function upon L-T4 replacement. This contrasts with the Tei Index, where both Subclinical Hypothyroidism and Congenital Hypothyroidism subgroups showed significant improvements (decreases in Tei Index). This suggests that while L-T4 broadly improves cardiac performance across different types of hypothyroidis, its effect on the gross measure of LVEF might be most pronounced in overt cases where cardiac dysfunction is likely more severe at baseline. The Post-Thyroidectomy subgroup for LVEF (18) showed a non-significant mean increase (MD = -1.6352), mirroring the non-significant increase in Tei Index for this group. Similarly, the Subclinical Hypothyroidism subgroup for LVEF (16) showed a negligible, non-significant increase (MD = -0.0140), whereas for Tei Index, this group showed a significant improvement. This highlights a potential difference in sensitivity: Tei Index might be a more sensitive indicator of subtle cardiac dysfunction and its reversal in conditions like subclinical hypothyroidism where LVEF may already be preserved or show minimal changes. Furthermore, the test for subgroup differences by disease type for LVEF approached significance (Q = 6.78, p=0.0791), suggesting that the varying cardiac responses to L-T4, while numerically distinct, did not reach statistical significance between disease types at this level of analysis. This is in contrast to the statistically significant subgroup differences observed for the LV Tei Index across disease types (Q = 6.99, p=0.0304), further emphasizing that the type of underlying thyroid dysfunction critically influences the therapeutic impact on overall myocardial performance (Tei Index), perhaps more distinctly than on LVEF alone. Finally, the subgroup analysis by follow-up period for LVEF revealed a statistically significant increase only at the 12-week follow-up [MD = -7.9300, Chou et al. (15)], coinciding with the Overt Hypothyroidism subgroup result (as Chou et al. is the sole study in both). Other follow-up periods for LVEF showed non-significant changes. This again suggests that the improvement in LVEF might be more time-dependent or specifically pronounced in overt cases. The overall lack of statistical significance in subgroup differences by follow-up period for LVEF (p=0.1300) indicates that the duration of treatment, on its own, did not consistently lead to differential LVEF responses across the meta-analysis.

The analyses of Mitral E velocity and Mitral A velocity shared identical statistical outcomes for the overall effect and subgroup differences, both showing an overall statistically significant increase after L-T4 therapy. The Congenital Hypothyroidism subgroup particularly demonstrated a statistically significant increase in both E and A velocities, while the Subclinical Hypothyroidism and Post-Thyroidectomy subgroups showed non-significant changes. The significant subgroup differences (p=0.0304) for both E and A velocities indicate that L-T4 differentially impacts these early and late diastolic filling parameters based on the patient’s underlying thyroid condition. An increase in both E and A velocities could reflect improved ventricular relaxation and/or increased left atrial contribution to filling in these conditions, though detailed interpretation requires consideration of E/A ratio and other diastolic parameters.

In stark contrast, the E/A ratio demonstrated no statistically significant change overall following L-T4 intervention. Furthermore, and notably different from the LV Tei Index and LVEF, there was no heterogeneity across studies for the E/A ratio, and no statistically significant differences between subgroups based on disease type. This suggests that despite observed improvements in overall myocardial performance (Tei Index) and, in some cases, systolic function (LVEF), L-T4 therapy does not consistently or significantly alter the fundamental ratio of early to late diastolic filling as represented by the E/A ratio. This may imply that the E/A ratio is less sensitive to the subtle improvements in diastolic function conferred by L-T4, or that such improvements are primarily reflected in other, more advanced diastolic parameters not explicitly analyzed here for the E/A ratio.

For subclinical hypothyroidism, L-T4 treatment consistently demonstrates beneficial effects on several cardiovascular surrogate markers. Studies indicate improvements in endothelial function, evidenced by increased Flow-Mediated Dilation (1), and reductions in both systolic and diastolic blood pressure (2). Furthermore, L-T4 therapy has been shown to favorably modify lipid profiles by lowering total cholesterol and low-density lipoprotein cholesterol (4), and reducing oxidative stress (19), all of which are crucial in mitigating cardiovascular risk. These findings suggest that L-T4 can address several underlying mechanisms contributing to cardiovascular pathology in SCH patients.

However, the benefits of L-T4 intervention are not uniformly observed across all cardiovascular parameters or patient demographics. While some studies suggest broad cardiovascular benefits, the meta-analysis by Swaid B et al. (1) did not find a significant reduction in Carotid Intima-Media Thickness (CIMT), a marker of arterial wall thickening. This highlights that L-T4’s impact might be more pronounced on functional aspects of the vasculature (like endothelial function and blood pressure) rather than directly reversing established structural changes. Moreover, for older adults (aged 65 and above) with SCH, Holley M et al. (20) concluded that L-T4 use was not associated with a significant difference in overall cardiovascular outcomes, contrasting with potential mortality benefits observed in younger SCH adults (21). This age-dependent response underscores the importance of patient stratification in treatment decisions.

Beyond cardiovascular health, L-T4 treatment’s efficacy and considerations extend to specific patient populations and administration protocols. For instance, in pregnant women with SCH, the benefits of L-T4 on pregnancy outcomes, such as reducing miscarriage and preterm birth, have been debated across studies, with some finding benefits (22, 23) and others not (24). The impact of different L-T4 administration regimens (e.g., weekly vs. daily, or liquid vs. tablet) on thyroid hormone levels remains inconclusive due to insufficient evidence and high heterogeneity in current research (25, 26). While L-T4 is effective in normalizing TSH levels, its broader impact on quality of life or the alleviation of residual symptoms in hypothyroid patients, or its role in conditions like depression or migraines, often shows mixed or non-significant results in meta-analyses (27, 28). These varying outcomes across different patient groups and clinical endpoints emphasize the need for individualized treatment approaches and further high-quality research, particularly for long-term and patient-centered outcomes.

The mechanistic understanding of L-T4’s influence on cardiac function is multifaceted. Thyroid hormones are known to exert direct effects on myocardial contractility and relaxation through genomic and non-genomic pathways. At a molecular level, L-T4, primarily after deiodination to L-T3, binds to thyroid hormone receptors in cardiomyocytes, regulating the expression of genes involved in cardiac structure and function. This includes genes encoding for sarcoplasmic/endoplasmic reticulum Ca2+ ATPase, α-myosin heavy chain, and β-myosin heavy chain, influencing calcium handling and contractile protein isoform expression, respectively (29). For instance, L-T4 treatment has been shown to restore normal left ventricular diastolic strain rate in overt hypothyroidism, suggesting an improvement in myocardial relaxation properties (30). The observed increases in Mitral E and Mitral A velocities in this meta-analysis are consistent with improved early and late diastolic filling, further supporting the role of L-T4 in enhancing myocardial relaxation. Beyond direct myocardial effects, L-T4 also influences the cardiovascular system through systemic hemodynamic changes, including a reduction in systemic vascular resistance and an increase in cardiac output, which can indirectly affect left ventricular loading conditions and performance. Studies have demonstrated that maternal hypothyroidism can significantly impact fetal cardiac output, with L-T4 intervention potentially mitigating these effects (31). This is particularly relevant given the findings in the congenital hypothyroidism subgroup, where a significant decrease in LV Tei Index and a significant increase in Mitral E and A velocities were observed. These results underscore the importance of early and adequate L-T4 replacement in conditions where thyroid hormone deficiency is established early in development.

Furthermore, L-T4 therapy has been shown to improve various cardiovascular risk factors, such as blood pressure, lipid profiles, and inflammatory markers, as highlighted in the introduction (2–5). These systemic improvements could contribute indirectly to better cardiac mechanics over time. The role of angiopoietin-like proteins as emerging cardiovascular biomarkers that are altered in hypothyroidism and improve with L-T4 treatment also suggests broader systemic and endothelial effects that could contribute to improved cardiac function (32). The varying responses observed across different disease types and follow-up durations suggest that the cardiac benefits of L-T4 may be dependent on the severity and duration of the thyroid hormone deficiency, as well as the specific cardiac parameters being assessed. For instance, the statistically significant improvement in LVEF in the overt hypothyroidism subgroup at 12 weeks (15), but not in other subgroups or overall, indicates that more severe thyroid hormone deficiencies might lead to more pronounced, and therefore more readily reversible, cardiac dysfunction with L-T4 intervention. Studies on idiopathic dilated cardiomyopathy have also shown that short-term L-T4 treatment can significantly improve LV mechanics and functional status (33), further supporting its role in improving cardiac contractility. Conversely, in mild subclinical hypothyroidism in older adults, L-T4 may not significantly alter cardiac systolic or diastolic function (34), which aligns with the non-significant overall findings for LV Tei Index and LVEF in this meta-analysis. The inclusion of studies exploring L-T4 and L-T3 combination therapy (18, 35) also provides insight into potential differential effects. While our meta-analysis did not find a statistically significant difference between L-T4 monotherapy and combination therapy for LV Tei Index or LVEF, individual studies suggest that combined therapy may improve left atrial volume index and atrial conduction times in patients with low T3 levels despite L-T4 treatment (35). This highlights the ongoing need for larger studies to explore the optimal dosing and combination strategies for various cardiac outcomes. Animal studies have also explored the cardioprotective effects of other thyroid hormone derivatives like 3,5-diiodothyronine (3,5-T2), showing improvements in heart rate variability and protection against ischemia-reperfusion injury, suggesting a broader range of therapeutic possibilities for thyroid hormone mimetics beyond standard L-T4/L-T3 therapies (36).

The influence of hormonal fluctuations over a lifetime represents a critical dimension for optimizing L-T4 therapy. As highlighted in research on cerebrovascular health, the profound shifts in sex hormone levels—such as the decline in estrogen during menopause in women—can significantly alter vascular pathophysiology and dynamics (37). This varying hormonal milieu suggests that the cardiovascular response to, and thus the requirement for, a fixed L-T4 dose is not static but differs by sex and across life stages (e.g., pre- vs. post-menopause). Consequently, L-T4 dosage for an individual may need dynamic adjustment with aging and major hormonal transitions, underscoring the need for a patient-tailored approach that moves beyond reliance on TSH alone to consider the patient’s evolving endocrine context.

The compilation of data from different centers in this meta-analysis also prompts consideration of alternative monitoring modalities. While echocardiography provided the functional cardiac data for this study, radionuclide imaging presents a potential tool for assessing therapeutic response at a metabolic level. Specifically, for monitoring systemic effects of L-T4 therapy, techniques such as iodine-123 scintigraphy could be employed to assess residual thyroid tissue function and turnover, while technetium-99m sestamibi scans might evaluate changes in myocardial perfusion and mitochondrial function—key aspects influenced by thyroid status (38). The variability observed in our echocardiographic results appears to be more population-specific than center-based, as evidenced by the consistent responses within distinct clinical subgroups (e.g., congenital vs. post-thyroidectomy) despite differing origins. This synthesis across centers strengthens the conclusion that the underlying etiology of hypothyroidism is a primary determinant of cardiac response, underscoring the necessity of tailoring both monitoring strategies and clinical expectations to the individual patient’s pathophysiology.

Building upon the findings of this meta-analysis, future clinical trials are warranted to refine L-T4 treatment strategies and optimize dose adjustment protocols. Specifically, randomized controlled trials should be designed to investigate whether therapy guided by sensitive echocardiographic parameters, such as the Tei index, can yield superior cardiac outcomes compared to the current standard of care which relies primarily on serum TSH levels. These trials should prioritize high-risk populations identified in our analysis, including patients with overt hypothyroidism where LVEF improvements were noted, and those with subclinical hypothyroidism who demonstrated enhanced global myocardial performance. Furthermore, trials should explore dynamic dosing algorithms that integrate factors beyond TSH, such as patient age, sex, and life-stage specific hormonal status, to determine if a more personalized approach can more effectively reverse cardiac dysfunction and improve long-term cardiovascular prognosis. The potential role of combination therapy with L-T3 in patients with persistent symptoms or specific cardiac phenotypes, such as impaired diastolic function, also remains a critical area for future investigation.

## Limitations

Despite the valuable insights gained, this meta-analysis has several limitations. First, the relatively small number of included studies and observations for certain endpoints may limit the statistical power to detect subtle but clinically meaningful effects, particularly within subgroups. Second, the heterogeneity in study designs (RCTs vs. cohort studies), patient populations (varying disease etiologies and severities, including a mix of adult and neonatal patients), and L-T4 dosages/regimens across studies could introduce variability and complicate direct comparisons, despite the use of random-effects models and subgroup analyses. The wide confidence intervals observed for some subgroup effects reflect this inherent variability. Third, the quality of echocardiographic measurements can vary between centers, and while efforts were made to extract comprehensive data, subtle methodological differences in image acquisition and analysis could exist across studies. Fourth, the follow-up durations varied considerably, ranging from 2 weeks to 12 months, making it challenging to ascertain long-term effects or the optimal duration for observing cardiac improvements. Lastly, while some studies included blinding, the overall risk of bias assessment indicated unclear or high risk for certain domains in several included studies, potentially affecting the reliability of their findings and the overall meta-analysis. Future research with larger, well-designed randomized controlled trials focusing on specific patient populations and standardized echocardiographic endpoints are warranted to further clarify the cardiac effects of L-T4 intervention.

## Conclusion

This meta-analysis demonstrates that L-T4 intervention impacts various echocardiographic parameters of left ventricular function, with significant improvements observed in the LV Tei Index in subclinical and congenital hypothyroidism, and in LVEF in overt hypothyroidism. Mitral E and A velocities also showed overall improvements, with significant variability across disease types. However, the E/A ratio showed no significant change. These findings underscore the differential cardiac responses to L-T4 therapy based on the underlying thyroid condition and follow-up duration, suggesting that L-T4 replacement contributes to improved myocardial performance and diastolic filling in specific hypothyroid contexts.

## Data Availability

The raw data supporting the conclusions of this article will be made available by the authors, without undue reservation.
